# Editorial: Nuclear Medicine in the Context of Personalized Medicine

**DOI:** 10.3389/fmed.2020.00252

**Published:** 2020-06-09

**Authors:** Jacques Barbet, Myriam Bernaudin, Pierre Payoux, Francesco Cicone, Marie-Hélène Gaugler, Françoise Kraeber-Bodéré

**Affiliations:** ^1^GIP Arronax, Saint-Herblain, France; ^2^Université de Normandie, UNICAEN, CEA, CNRS, ISTCT/CERVOxy Group, GIP CYCERON, Caen, France; ^3^Service de Médecine Nucléaire, CHU de Toulouse, Toulouse, France; ^4^ToNIC, Toulouse NeuroImaging Center, UMR1214 Inserm, Toulouse, France; ^5^Department of Experimental and Clinical Medicine, “Magna Graecia” University of Catanzaro, Catanzaro, Italy; ^6^Department of Nuclear Medicine and Molecular Imaging, Lausanne University Hospital, Lausanne, Switzerland; ^7^Université de Nantes, CNRS, Inserm, CRCINA, Nantes, France; ^8^Université de Nantes, CHU de Nantes, ICO Gauducheau, CNRS, Inserm, CRCINA, Nantes, France

**Keywords:** PET, theranostics, radionuclide therapy, solid tumors, hematological malignancies

Nuclear Medicine has been at the heart of theranostics even before the term was coined. It provides effective tools for precision medicine particularly with positron emission tomography (PET), and radioligand therapy. It allows the investigation of phenotypes and functions in all areas of medicine and provides innovative tools to kill cancer cells. This Research Topic for Frontiers in Medicine focuses on the role of Nuclear Medicine in the context of Personalized Medicine.

Three reviews reported the growing interest of PET in onco-hematological diseases. Jamet et al. have demonstrated the interest of 2-[^18^F]fluoro-2-deoxy-D-glucose ([^18^F]FDG) PET in the management of patients with multiple myeloma (MM). [^18^F]FDG-PET is highly sensitive and specific for bone lesions detection at baseline. The presence of extra-medullary disease, the number of bone focal lesions and the maximum standardized uptake value [SUVmax] have independent pejorative prognostic value on progression-free survival and overall survival. For therapy response assessment, [^18^F]FDG-PET is considered as the reference imaging technique. [^18^F]FDG-PET and bone marrow flow cytometry are complementary for detection of minimal residual disease before maintenance therapy. New PET tracers such as [^11^C]methionine, choline or acetate,[^68^Ga]Ga-pentixafor, which targets CXCR4, and immuno-PET targeting CD138 and CD38, also show promising results.

As discussed by Bailly et al., [^18^F]FDG-PET changed response assessment and therapy strategy in diffuse large B-cell lymphoma (DLBCL) and Hodgkin lymphoma. [^18^F]FDG-PET might also have a significant impact in the management of mantle cell lymphoma (MCL). [^18^F]FDG-PET at baseline in MCL patients has good sensitivity for staging and SUVmax provides prognostic information. They conclude that [^18^F]FDG-PET results should be integrated in the definition of MCL treatment strategy to identify patients who might benefit from more intensive therapy.

The specificity of [^18^F]FDG uptake has been questioned. Thus, new tracers such as [^18^F]Fludarabine, a nucleoside analog, have been developed. Early results with [^18^F]Fludarabine have been reviewed by Barré et al.. Favorable preclinical results in murine models (follicular and central nervous system lymphoma, MM) have prompted a “first in man” study. In DLBCL patients, increased uptake was observed in sites considered abnormal by CT and [^18^F]FDG. In chronic lymphocytic leukemia patients, increased uptake coincided with lymph-nodal sites expected to be involved by the disease. [^18^F]Fludarabine uptake was also high in the spleen and bone marrow. No uptake was observed in the cardiac muscle and brain. They conclude that [^18^F]Fludarabine might correctly quantify the disease burden, in the presence of confounding inflammatory processes.

Monoclonal antibody (mAb)-based therapies and immunotherapy have experienced considerable growth in cancer management. Labeled mAbs also show promise for theranostics. While PET can be performed using directly radiolabeled mAbs, pretargeting improves imaging contrast. The development of pretargeted immuno-PET in tumors expressing carcinoembryonic antigen (CEA; CEACAM5) has been reviewed by Bodet-Milin et al., focusing on medullary thyroid carcinoma. They conclude that pretargeted PET imaging has a high potential for antibody-based diagnostics and theranostics.

Immunotherapy relies on *in situ* activation or inhibition of immune cells or on the administration of immune cells selected, activated, or transformed *ex vivo*. It is most important to delineate, by *in vivo* imaging, the distribution, activation and migration of immunologically active cells. Methods designed to monitor the fate of these cells and to define their immunological status have been reviewed by Perrin et al., focusing on cell tracking in cancer immunotherapy, particularly CAR (Chimeric Antigen Receptor) T-cell therapy, and on its potential impact on these new therapeutic modalities.

Severe hypoxia, frequent in glioblastoma multiforme, is associated with resistance to ionizing radiation. It contributes to treatment failures after external-beam radiation therapy (EBRT). It would be logical to deliver higher radiation doses to hypoxic tumor regions. This calls for the delineation of hypoxic zones as examined by Gérard et al. Preliminary *in silico* studies investigate the conversion of hypoxia maps into dose-distribution objectives for EBRT dose painting in view of future clinical trials.

The ability to monitor the distribution of radioactivity inside the body is a major advantage of nuclear medicine procedures. Rhenium-188 (^188^Re) is an attractive candidate for therapy and has a favorable gamma emission for imaging purposes. It is readily extracted from ^188^W/^188^Re generators and exhibits chemical properties similar to those of technetium-99m, which might constitute an additional, purely diagnostic companion radionuclide. The development of radiopharmaceuticals based on ^188^Re, including peptides, mAbs, and particles has been reviewed by Lepareur et al., demonstrating that ^188^Re is a cost-effective alternative for routine clinical use.

In neuroscience, carbon-11 or fluorine-18 can be used to label molecules that cross the blood brain barrier, the latter being considered preferable for clinical use. Molecular imaging has focused on receptors, neurotransmitter transporters, and other proteins and PET and SPECT biomarkers have become indispensable for clinical research and the selection of treatment options in a number of pathologies, notably neurodegenerative diseases. They can be used for assessing patients' eligibility for new treatments, or for treatment follow-up. The review by Beaurain et al. describes some radiotracers used in neuroscience according to their target: dopaminergic, cholinergic or serotoninergic systems, β-amyloid plaques, tau protein, neuroinflammation, glutamate or GABA receptors, or α-synuclein.

Targeting the membrane dopamine transporter (DAT) proves useful in the follow-up and treatment assessment of brain diseases. Carbon-11 and fluorine-18 labeled tracers have been derived from the chemical structure of cocaine. The review by Chalon et al. focuses on the development of one such tracer, LBT-999. [^18^F]LBT-999 proved capable of exploring *in vivo* the localization of DAT at the dopaminergic nerve endings as well as at the mesencephalic cell bodies in lesion-induced rat models of Parkinson's disease. Recent clinical data demonstrated the efficiency of [^18^F]LBT-999 in the diagnosis of Parkinson's disease.

To complete this overview, risk management has been discussed by Lonceint et al. as a major concern for health organizations. In hospitals, medical personnel may be exposed to ionizing radiation and the highest doses (up to a few mSv) are recorded in nuclear medicine departments. The review aims at understanding the attitude of health professionals toward the risks of exposure and how they combine patient care with self-protection. The coexistence of care and radiation protection logics was shown to be a source of contradictions for nuclear medicine professionals and of differences in risk regulation strategies according to occupational groups.

## Author Contributions

JB wrote the editorial, which was revised, proofed, and accepted by all authors.

## Conflict of Interest

The authors declare that the research was conducted in the absence of any commercial or financial relationships that could be construed as a potential conflict of interest.

